# Foods with added fiber improve stool frequency in individuals with chronic kidney disease with no impact on appetite or overall quality of life

**DOI:** 10.1186/1756-0500-6-510

**Published:** 2013-12-05

**Authors:** Younis A Salmean, Gordon A Zello, Wendy J Dahl

**Affiliations:** 1 Food Science and Human Nutrition, University of Florida, 359 FSHN Building Newell Drive, Gainesville, FL 32611, USA; 2 College of Pharmacy and Nutrition, University of Saskatchewan, 110 Science Place, Saskatoon SK S7N 5C9, Canada

**Keywords:** Chronic kidney disease, Fiber, Quality of life, Gastrointestinal function, Appetite, GSRS, SNAQ, ESS

## Abstract

**Background:**

Fiber intake may be low in individuals with chronic kidney disease (CKD) due to diet restriction and/or poor appetite associated with uremic symptoms, contributing to constipation and reduced quality of life. This report describes the effects of foods with added fiber on gastrointestinal function and symptoms, clinical markers, and quality of life in CKD patients.

**Findings:**

Adults with CKD (n = 15; 9 F, 6 M; 66 ± 15 y) were provided with cereal, cookies and snack bars without added fiber for 2 weeks, followed by similar foods providing 23 g/d of added fiber for 4 weeks, to incorporate into their usual diets. Participants completed the Kidney Disease Quality of Life (KDQOL-36) questionnaire, the Simplified Nutritional Appetite Questionnaire (SNAQ) and the Epworth Sleepiness Scale (ESS) bi-weekly, the Gastrointestinal Symptom Rating Scale (GSRS) weekly, and daily stool frequency and compliance. Control and intervention serum cholesterol and glucose were assessed. Providing 23 g/d of added fiber increased stool frequency (1.3 ± 0.2 to 1.6 ± 0.2 stools/d; P = 0.02), decreased total cholesterol (175 ± 12 to 167 ± 11 mg/dL; P = 0.02) and improved TC:HDL ratio (4.0 ± 0.3 to 3.7 ± 0.2; P = 0.02). GSRS and SNAQ scores did not change, but SNAQ scores suggested poor appetite in 7 participants with or without added fiber. KDQOL *Mental Health Composite* decreased from 53 ± 2 to 48 ± 2 (P = 0.01) while *Physical Health Composite* increased from 31 ± 2 to 35 ± 3 (P = 0.02), with no change in overall QOL. The ESS score decreased from 10 ± 1 to 8 ± 1 (P = 0.04).

**Conclusion:**

Consuming foods with added fiber may be an effective means of increasing fiber intakes, improving stool frequency, and lipid profile in individuals with CKD.

**Trial registration:**

ClinicalTrials.gov, # NCT01842087

## Introduction

The fiber intake of Americans falls dramatically short of the amount recommended for good health, with the majority of adults and children averaging half of the recommendation [1]. The consumption of fiber has been decreasing over the past decade and is lower in African-American adults, a population at increased risk of developing CKD [2] and kidney failure [3]. Patients with advanced CKD may need to limit foods naturally rich in fiber such as whole grains, legumes and certain fruits and vegetables due to their phosphorous and/or potassium contents [4]. Poor appetite, commonly experienced by individuals with uremia due to CKD, may also negatively impact fiber intake. Thus, individuals with CKD may have fiber intakes lower than that of the healthy population.

Dietary fiber has been shown to confer several health benefits related to cardiovascular disease and diabetes. Isolated fibers, specifically viscous fibers, are known to attenuate blood glucose response and to lower LDL and total cholesterol [5]. Whole foods, such as pulses, that are high in dietary fiber also have been shown to demonstrate reductions in glycemia [6] and to improve cholesterol profile [7,8]. Diabetes and hypertension are the two leading causes of renal failure accounting for about 60% of all new cases [3], while cardiovascular disease is the most likely cause of death [9]. Given that many with CKD have diabetes and all are at risk for cardiovascular disease, low fiber intake may be particularly detrimental in this population.

Individuals with CKD experience a significant decline in quality of life with disease progression [10]. This may be attributed mainly to uremia, which includes symptoms such as anorexia, constipation, nausea, fatigue and decreased mental acuity [11]. Increases in the number and severity of symptoms lead to a more pronounced decline in quality of life. These uremic symptoms are assumed to be mainly the result of accumulation of organic molecules that are normally cleared by the kidneys [11]. Studies have shown that supplementing fiber leads to decreased uremic molecules [12-14]; hence fiber may positively impact quality of life by reducing uremia, constipation in particular. However, certain fibers, such as oligofructose, may reduce energy intake, possibly through reduced appetite [15]. As individuals with CKD are at risk for malnutrition, decreased appetite may be a potential risk with added fiber.

Foods with added fiber may be of benefit to individuals with CKD. We hypothesized that incorporating commercially-available foods with added fiber into the diets of individuals with CKD would lead to improved gastrointestinal function and quality of life, without impacting clinical markers and symptoms. This study was conducted according to the guidelines laid down in the Declaration of Helsinki and all procedures involving human subjects were approved by the University of Florida’s Institutional Review Board 1 (IRB#16-2010). Written informed consent was obtained from all subjects. This study is registered with ClinicalTrials.gov, registration # NCT01842087.

## Findings

A six-week, single-blind, rolling admission study was carried out. As reported previously, nephrology patient charts (n = 270) were screened for eligible participants using the inclusion criteria: > 18 y, eGFR of ≤ 50 mL/min/1.73 m^2^ (stage 3, 4 and 5 but not on dialysis), no acute kidney injury or glomerulonephritis, and no immunosuppressant medications [16]. Participants with CKD were provided with control foods (cookies, snack bars and breakfast cereal) containing < 2 g/day of fiber for 2 weeks, followed by similar foods providing 23 g/day (pea hull, inulin and resistant corn dextrin) for 4 weeks, to incorporate into their usual diets. Four weeks was chosen for the length of the fiber intervention to allow for potential changes in gut microbiota, and its predicted impacts on previously reported outcomes [16], whereas the control was limited to 2 weeks to minimize participant burden. Participants completed the Kidney Disease Quality of Life (KDQOL-36) questionnaire [17], the Simplified Nutritional Appetite Questionnaire (SNAQ) [18] and the Epworth Sleepiness Scale [19] bi-weekly at clinic visits, and the Gastrointestinal Symptom Rating Scale (GSRS) [20], weekly. A SNAQ score of ≤ 14 indicates poor appetite and significant risk of at least 5% weight loss within six months. The Epworth Sleepiness Scale was used to assess participants likelihood of daytime sleepiness. Scores ranging from 10 to 12 indicate a boarderline risk of daytime sleep propensity, while a range of 12 to 24 indicates an abnormal tendency. In addition, participants recorded daily stool frequency and compliance. Fasting serum glucose and cholesterol were assessed by clinically standardized methods on blood samples collected on days 1 and 14 of the control period and days 28 and 42 of the intervention period. Paired t-test was used for comparisons of parameters and scores for each test between periods. Significance was considered to be p < 0.05. All data are presented as mean ± SE.

Seventeen individuals with stage 3 to 5 CKD (66 ± 15 y) consented to participate in the study. Sixteen enrolled and 15 completed the study. Of those completing the study, four patients were in stage 3, 10 were in stage 4, and one was in stage 5 (pre-dialysis). Eleven participants were White and 4 were African American, with 9 females and 6 males. With a daily intake of 16.8 g of fiber [16] of the 23 g/d offered, daily stool frequency increased with the fiber intervention from 1.3 ± 0.2 to 1.6 ± 0.2 stools per day (P = 0.02). There were no changes in fasting blood glucose (Table 1). Total cholesterol (TC) decreased from 175 ± 12 to 167 ± 11 mg/dL (P = 0.02) and a strong trend was found for decreased LDL cholesterol (100 ± 8 to 93 ± 7 mg/dL; P = 0.05). TC:HDL ratio declined from 4.0 ± 0.3 during control to 3.7 ± 0.2 during the fiber intervention (P = 0.02) (Table 1). No significant changes were found in the average SNAQ scores with the fiber intervention (Table 1). Epworth Sleepiness Scale score decreased from 10 ± 1 at control to 8 ± 1 with the fiber intervention (P = 0.04) (Table 1). The GSRS scores indicated low gastrointestinal distress and did not change from control (23 ± 1) to fiber intervention (22 ± 1). There were no changes in the KDQOL-36™ overall mean score or with the *Symptom/Problem List*, *Effects of Kidney Disease* and *Burden of Kidney Disease* subscales throughout the study (Table 1). However, the *Physical Component Summary* subscale mean score increased from 31 ± 2 during control to 35 ± 3 during fiber intervention (p = 0.02). The mean score for the *Mental Component Summary* subscale was significantly lower during intervention (48 ± 2) compared to control (53 ± 2) (P = 0.01). Comparisons between males and females showed no significant differences (data not reported).

**Table 1:** Clinical markers, symptoms and quality of life

	**Control period (no fiber added)**	**Intervention period with added fiber**	**P value**
Weight (kg)	89 ± 6	90 ± 6	NS
**Clinical markers**			
Glucose mg/dL	136 ± 29	146 ± 25	NS
LDL mg/dL	100 ± 8	93 ± 7	P = 0.05
HDL mg/dL	47 ± 4	47 ± 4	NS
Total CHOL mg/dL	175 ± 12	167 ± 11	P = 0.02
TG mg/dL	165 ± 21	154 ± 21	NS
CHOL:HDL ratio	4.0 ± 0.3	3.7 ± 0.2	P = 0.02
**KDQOL-36™**			
*Symptom/Problem list*	78 ± 3	80 ± 3	NS
*Effects of kidney disease*	84 ± 4	87 ± 3	NS
*Burden of kidney disease*	67 ± 6	73 ± 6	NS
*Physical component summary*	31 ± 2	35 ± 3	P = 0.02
*Mental component summary*	53 ± 2	48 ± 2	P = 0.01
Overall mean scores	63 ± 3	64 ± 3	NS
**Symptoms**			
SNAQ	14 ± 1	14 ± 1	NS
GSRS	23 ± 1	22 ± 1	NS
ESS	10 ± 1	8 ± 1	P = 0.04
Stool frequency	1.3 ± 0.2	1.6 ± 0.2	P = 0.02

Data are means ± SE. Values are compared using paired t-test and significance is determined at P < 0.05.

Constipation is a common symptom of uremia contributing to decreased quality of life in individuals with advanced CKD. Foods with added fiber may provide a remedy. With the provision of 23 g/d of added fiber, participants in the current study improved their fiber intakes, consuming an additional 16.8 g, on average, of added fiber, in addition to the 9.7 g fiber in their background diet [16]. The majority of participants achieved their Adequate Intake for fiber [16]. Stool frequency increased, due in part to the 4 g/d of insoluble pea hull fiber provided in the foods with added fiber. This finding is in agreement with a previous study where 4 g/d of pea hull fiber improved stool frequency in elderly residing in a long term care home [21]. The inulin may have also contributed to increased stool frequency as this effect has been demonstrated in constipated elderly [22].

A concern with providing the foods with added fiber was the additional available carbohydrate provided. However, no change in body weight or energy intake occurred over the six-week study [16]. Lipid profile improved with the fiber intervention, although no viscous fiber was provided and thus, the change may be due to the participants’ reduced intake of fat, as reported previously [16]. Given the significant cardiovascular risk of the CKD patient population, the improvements in TC and LDL, although unexpected, were positive findings.

The fiber intervention had little impact on symptom scores with the exception of sleepiness. The change in ESS score suggests a drop from borderline risk of daytime sleepiness to normal. Participants reported an average SNAQ score of 14 during control with seven participants at risk for future weight loss due to poor appetite. During control, there were five participants with average scores of 10 or higher compared to only three participants during intervention period. As malnutrition is of concern in the later stages of CKD [23], the impact of added fiber on appetite justifies further investigation.

CKD patients, with creatinine clearance of <60 mL/min/1.73 m^2^, report lower physical function independent of age, sex, and other confounding factors [24]. In dialysis patients, both the *Physical* and *Mental Components* of the KDQOL-36 were consistent predictors of hospitalizations and mortality rates with each point increase in either component reducing mortality relative risk by 2%, and hospitalization relative risk by 2% and 1%, respectively [25]. Thus, the 4-point improvement in the *Physical Component Summary* and the decrease in *Mental Component Summary* reported in this study may be of clinical significance. The improved physical and functional health may be explained by the increased stool frequency as it has been reported that quality of life is significantly impacted by constipation [26]. The increase in the *Physical Component Summary* also may be due to the improved kidney function reported previously [16]. The *Mental Component Summary* was lower during the intervention period when compared to control. This decline in mean score may be explained by those participants who did not respond to the intervention as five out of seven participants who reported lower scores during intervention were non-responders. It has also been suggested that changes in *Mental Component Summary* are unlikely to be correlated to kidney function among those in stage 4 and 5 of the disease [10], but may be associated with variables that were not explored.

This study had limitations. The sample of CKD patients was diverse, including both White and African-Americans in CKD stages 3, 4 and 5, therefore too small to allow for statistical comparisons among race and stage of disease. The results of this study are considered pilot data and thus, further research is needed to confirm the findings presented.

Fiber intakes of the general population fall far below the recommendation and those with CKD may be at increased risk from this shortfall. Foods with added fiber, specifically those that are conducive to the restrictions of a renal diet, may provide health benefits to those with CKD, particularly improved regularity, lipid profile and the physical/functional aspects of quality of life.

## Abbreviations

CKD: Chronic kidney disease; LDL: Low density lipoprotein; TC: Total cholesterol.

## Competing interests

The study was funded by the Saskatchewan Pulse Growers, Saskatoon, Canada. The authors have no other competing interests.

## Authors’ contributions

YAS refined the study design, carried out the study, collected the data, performed the statistical analysis and assisted with drafting the manuscript. WJD and GAZ initiated the study by proposing the original study and securing funding for the work. WJD was responsible for the coordination of the study and drafting the manuscript. GAZ assisted with drafting the manuscript. All authors read and approved the final manuscript.
